# Coexisting in a Crowded Field: A 10-year Comparison of Procedural Volumes of Plastic Surgeons and Other Surgical Specialties in the United States

**DOI:** 10.1055/a-2731-4559

**Published:** 2026-01-30

**Authors:** Ethan L. MacKenzie, Doruk Orgun, Kasey Leigh Wood Matabele, Samuel O. Poore

**Affiliations:** 1 Division of Plastic and Reconstructive Surgery, University of Wisconsin School of Medicine and Public Health, Madison, Wisconsin, United States; 2 Department of Plastic and Reconstructive Surgery, The Jikei University School of Medicine, Minato-ku, Tokyo, Japan; 3 Division of Plastic and Reconstructive Surgery, University of Rochester Medical Center, Rochester, New York, United States

**Keywords:** specialty, procedures, surgeries, complications, tissue

## Abstract

**Background:**

Plastic surgery has broad overlap with other surgical subspecialties. Regarding procedures provided by both plastic surgery and other surgical specialties in the United States, the changes in plastic surgical case volume over time have not been previously investigated.

**Methods:**

Select common procedure terminology (CPT) codes from an array of areas of practice including breast reconstruction, hand, adult craniofacial, peripheral nerve, microsurgery, and ventral hernia were extracted from the American College of Surgeons National Surgical Quality Improvement Program (ACS-NSQIP) from 2010 to 2020. Case numbers, operative times, and complication rates among surgical subspecialties were compared. Aesthetic surgery cases were excluded as they are not well captured by the NSQIP database.

**Results:**

A total of 128,545 procedures were included. In 2010 and 2020, plastic surgeons performed 83.9% versus 89.3% of reconstructive breast ( *p* < 0.001), 5.4% versus 4.3% of hand ( *p* = 0.23), 33.6% versus 39.1% of adult craniofacial ( *p* = 0.28), 36.7% versus 29.5% of peripheral nerve ( *p* = 0.45), 9.3% versus 94.6% of microsurgical ( *p* = 0.25), and 0.4% versus 0.4% of ventral hernia procedures ( *p* = 0.99). Plastic surgery performed the majority of breast reconstruction and microsurgical procedures. Operative times and complication rates varied between specialties and were generally inversely proportional to the number of cases for included specialties.

**Conclusion:**

Plastic surgical involvement did not significantly decrease for any of the included procedural categories in ACS-NSQIP over the study period. Breast reconstruction and microsurgery remain areas of particular specialization with plastic surgeons performing the highest volume. Areas of shared practice suggest opportunities for collaboration.

## Introduction

Plastic surgery is an inherently innovative specialty that addresses a broad spectrum of conditions from head to toe in patients of all ages and comorbidity status. Plastic surgeons have contributed immensely to major advancements in surgery, such as with the development of free tissue transfer with microvascular anastomosis throughout the 1970s. 1 2 3 4 Other historical advancements by plastic surgeons include the anatomical description of component separation in ventral hernia repair, 5 biomechanical restoration in brachial plexus repair via functioning free muscle transfers, 6 and the development of digital replantation with the first report of replantation of a totally amputated thumb. 7 Moreover, the principles of skin grafting were essential in the early immunologic experiments that eventually gave rise to solid organ transplantation in humans. 8 9 It is therefore not surprising that the field of plastic surgery shares significant clinical domain with other specialties, with many procedures having been adopted by surgeons of different specialties. In some areas, surgical techniques have been refined by collaboration with other specialties such as in the case of complex hernia repair. Other areas, such as hand surgery, exist at the interface of multiple specialties, with each contributing to the field. Lastly, some areas such as microvascular head and neck reconstruction have recently begun to move away from the domain of plastic surgeons and into the practice of head and neck surgeons.

In a recent study we demonstrated that plastic surgeons, despite being a relatively small group that shares intellectual spaces with surgeons of other subspecialties, continue to contribute robustly to academic innovation with respect to publication volume and number of citations in breast and aesthetic literature. 10 However, at present there is a paucity of literature regarding how plastic surgeons perform in areas of shared practice with other specialties, especially with regards to case volumes. In this study, the American College of Surgeons National Surgical Quality Improvement Program (ACS-NSQIP) database was utilized to compare case volumes and patient outcomes for select non-aesthetic procedures among plastic surgery and other surgical specialties in the United States between 2010 and 2020.

## Methods

### Study Design

This study is a registry-based observational study of patients undergoing breast, hand, microsurgery, peripheral nerve, adult craniofacial, and ventral hernia surgeries in the United States between the years 2010 and 2020. Hospitals participating in the ACS-NSQIP are not responsible for the validity of the data analysis or the conclusions derived by the authors.

### Data Sources and Study Population

The ACS-NSQIP database is a publicly accessible source of over 150 prospectively gathered demographic, comorbid, preoperative, perioperative, and 30-day postoperative variables recorded by clinicians in a HIPAA-compliant manner. Only cases performed on the adult population are captured, and the specialties of dermatology and oral surgery, which share numerous procedures with plastic surgery, are not included. Cases are randomly selected for inclusion from participating hospitals that span the entire United States. Thus, collected data represent academic and community hospitals in both urban and rural locations but do not include data from freestanding surgery centers. We began by extracting all cases with select Current Procedural Terminology (CPT) codes recorded in the ACS-NSQIP from January 1, 2010 to December 31, 2020. These codes were chosen such that subsequent analyses would pertain to the following select areas of plastic surgical practice: breast, hand, adult craniofacial, brachial plexus, microsurgery, and open ventral hernia repair. This choice was based on the presence of professional organization representation or associated fellowship training. Even though purely aesthetic surgical procedures are important components of plastic surgical practice, these were not included since these procedures are better captured through insurance databases and are not in the scope of this study. Furthermore, since ACS-NSQIP does not include procedures performed by the specialty of dermatology, skin cancer treatment and reconstruction were not included in this study. Only cases with single CPT codes were included to avoid factors that would confound operative time or complications. Although all cases captured by given CPT code searches were included in demographic analyses, to evaluate the impact of only those specialties that robustly contribute to a given surgical area and to eliminate the confounding effect of potentially incorrect data entry, specialties performing fewer than 10 of a given case over the study period were excluded from further analysis. Case selection is summarized in Supplemental Fig. S1 .

### Statistical Analysis

Statistical analyses began with descriptive statistics to compare the frequency of involvement between various surgical specialties for selected cases. Case volumes over time within specialties were then compared using Chi-square tests to identify general trends.

For the comparison of operative times of given procedures among different surgical specialties, Mann-Whitney U tests (for the comparison of two specialties) and Kruskal-Wallis tests (for the comparison of more than two specialties) were used. In the case where more than two specialties were compared, further pairwise comparisons between plastic surgery and other specialties were performed by using Wilcoxon rank-sum tests supplemented by Bonferroni-Holm method for *p* -value adjustment.

Finally, Chi-square tests were performed to assess for any statistically significant differences in bleeding requiring transfusion, deep venous thrombosis (DVT), pulmonary embolism, wound infections, wound dehiscence, and readmission between surgical specialties. Initial data manipulations were performed in SAS 9.4 (Cary, NC) and R 4.2.0 (R Foundation, Vienna, Austria) and statistical analyses were run using SPSS 28.0 (IBM, NY). Statistical significance was set at α = 0.05.

## Results

### Breast

**Fig. 1:**
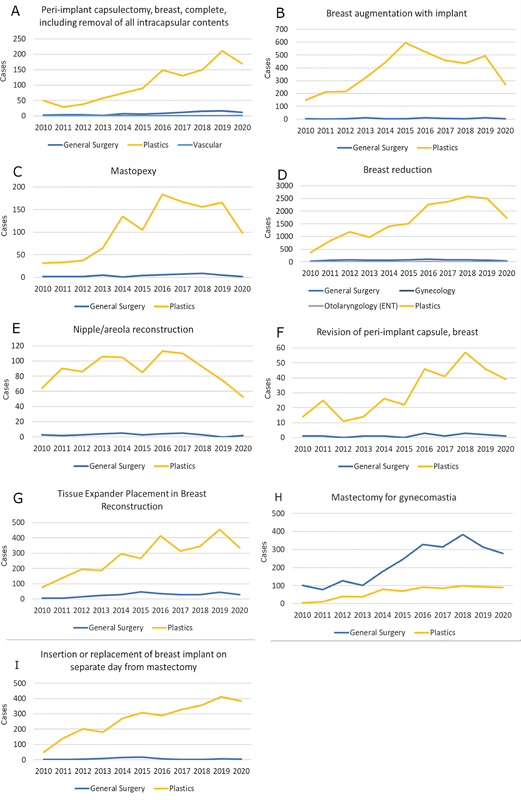
Year on year surgical volumes for selected breast procedures by operative specialty. The Y axis represents number of cases. ( **A** ) Peri-implant complete breast capsulectomy, including removal of all intracapsular contents. ( **B** ) Breast augmentation with implant. ( **C** ) Mastopexy. ( **D** ) Breast reduction. ( **E** ) Nipple/areola reconstruction. ( **F** ) Revision of peri-implant capsule, breast. ( **G** ) Tissue expander placement in breast reconstruction. ( **H** ) Mastectomy for gynecomastia. ( **I** ) Insertion or replacement of breast implant on separate day from mastectomy.

We reviewed 10 CPT codes accounting for 37,423 reconstructive breast procedures. Corresponding demographic data are shown in Supplemental Table S2 . An analysis comparing comorbidities between specialties is shown in Supplemental Table S1 . Approximately 89.0% ( *n* = 33,299) of all included procedures were performed by plastic surgeons with the exception of gynecomastia excisions, of which 77.4% were performed by general surgeons. Case volumes over the years are shown in Fig. 1 . In the years 2010 and 2020, plastic surgeons performed 83.9% versus 89.3% of selected reconstructive breast procedures ( *p* < 0.001). Yearly procedure volume increased for almost all procedures prior to 2020, during which procedure volumes decreased. One exception to this was nipple/areola reconstruction, with decreasing case volume starting in 2018.

With regards to operative times, only plastic surgeons and general surgeons performed high enough volumes of breast procedures to be included in the analysis. Table 1 presents median operative times for each breast procedure. Comparing plastic surgeons with general surgeons, median operative time in minutes were 152 versus 173 for breast reduction ( *p* < 0.01), 66 versus 74 for breast augmentation with implant ( *p* < 0.01), 81 versus 135 for immediate implant placement ( *p* < 0.01), 77 versus 92 for delayed implant placement ( *p* = 0.02), 77 versus 153 for tissue expander reconstruction ( *p* <0.01), 67 versus 111 for reconstruction revision ( *p* < 0.01), and 74 versus 45 for gynecomastia mastectomy ( *p* < 0.01).

**Table 1:** Median operative times for selected breast procedures compared across surgical specialties

Procedure	Specialty	Median time (minutes)	Interquartile range	*P* -value ^a^
**Mastectomy for gynecomastia**	Plastic surgery	74	48–112	<0.01
	General surgery	45	30–67	
**Mastopexy**	Plastic surgery	118	83–165	NS
	General surgery	104	77–138	
**Breast reduction**	Plastic surgery	152	111–195	<0.01
	General surgery	173	121–222	
**Breast augmentation with implant**	Plastic surgery	66	48–89	<0.01
	General surgery	74	59–109	
**Insertion of breast implant on same day of mastectomy (i.e., immediate)**	Plastic surgery	81	53–129	<0.01
	General surgery	135	92–215	
**Insertion or replacement of breast implant on separate day from mastectomy**	Plastic surgery	77	55–112	<0.01
	General surgery	92	64–142	
**Nipple/areola reconstruction**	Plastic surgery	59	36–90	NS
	General surgery	43	32–71	
**Tissue expander placement in breast reconstruction, including subsequent expansion(s)**	Plastic surgery	77	51–130	<0.01
	General surgery	153	90–228	
**Revision of peri-implant capsule, breast, including capsulotomy, capsulorrhaphy, and/or partial capsulectomy**	Plastic surgery	67	42–93	<0.01
	General surgery	111	78–159	
**Peri-implant capsulectomy, breast, complete, including removal of all intracapsular contents**	Plastic surgery	77	52–106	NS
	General surgery	87	56–105	

Note: ^a^ Mann-Whitney U tests were used for the comparison of two specialties.

Kruskal-Wallis tests were used for the comparison of more than two specialties. In this case, pairwise comparison between plastic surgery and other specialties were performed by Wilcoxon rank-sum tests with Bonferroni-Holm method to adjust *p* -values; *p* -values for the pairwise comparisons are reported in cases where significance tests for comparisons between plastic surgery and different specialties are contrasting.

Differences in complication rates for plastic surgery versus general surgery are presented in Table 2 and Supplemental Table S3 . Complication rates for delayed implant placement and implant revision procedures were inversely proportional to the case volumes performed by the specialties.

**Table 2:** Breast procedures for which there was a significant difference in complication rates between specialties

Procedure	Type of complication	Specialty	*N*	Complication *N* (%)	*P* -value
**Insertion or replacement of breast implant on separate day from mastectomy**	Readmission	Plastic surgery	2,929	59 (2.0%)	0.04
		General surgery	74	4 (5.4%)	
	Bleed requiring transfusion	Plastic surgery	2,928	0 (0%)	<0.01
		General surgery	74	1 (1.4%)	
	DVT	Plastic surgery	2,928	2 (0.07%)	<0.01
		General surgery	74	1 (1.4%)	
**Insertion of breast implant on same day of mastectomy**	Bleed requiring transfusion	Plastic surgery	1,120	0 (0%)	<0.01
		General surgery	133	1 (0.7%)	
**Revision of peri-implant capsule, breast, including capsulotomy, capsulorrhaphy, and/or partial capsulectomy**	Superficial infection	Plastic surgery	340	2 (0.6%)	0.09
		General surgery	14	1 (7.1%)	
**Peri-implant capsulectomy, breast, complete, including removal of all intracapsular contents**	Dehiscence	Plastic surgery	1147	5 (0.4%)	0.03
		General surgery	90	2 (2.2%)	

Note: Procedures for which there was no significant difference are not displayed. *N* for readmission and other complications differs slightly as readmission was not a variable collected in 2010.

### Hand

**Fig. 2:**
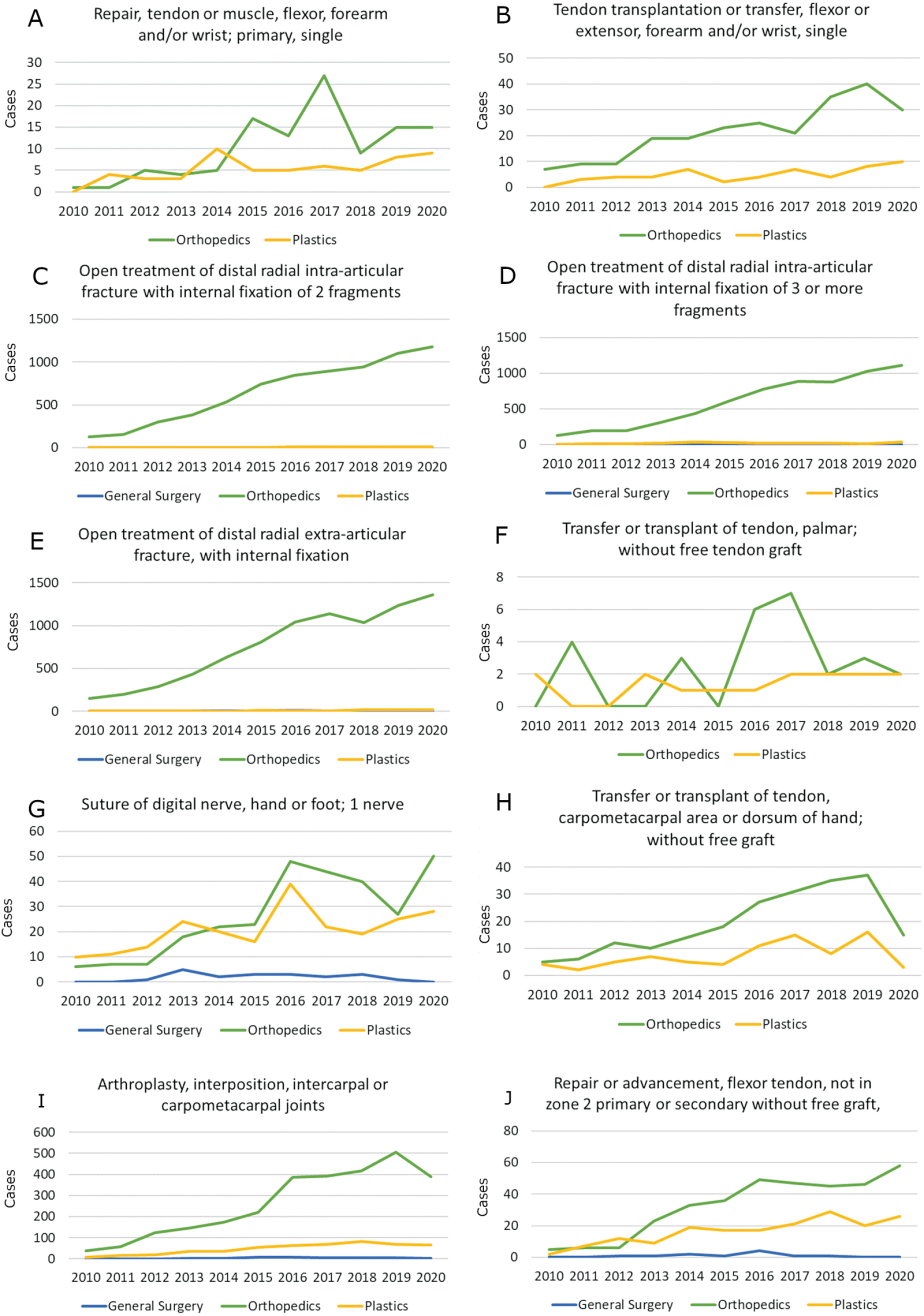
Year on year surgical volumes for selected hand procedures by operative specialty. The Y axis represents number of cases. ( **A** ) Repair, tendon or muscle, flexor, forearm and/or wrist; primary, single. ( **B** ) Tendon transplantation or transfer, flexor or extensor, forearm and/or wrist, single. ( **C** ) Open treatment of distal radial intra-articular fracture with internal fixation of two fragments. ( **D** ) Open treatment of distal radial intra-articular fracture with internal fixation of three or more fragments. ( **E** ) Open treatment of distal radial extra-articular fracture, with internal fixation. ( **F** ) Transfer or transplant of tendon, palmar; without free tendon graft. ( **G** ) Suture of digital nerve, hand or foot; one nerve. ( **H** ) Transfer or transplant of tendon, carpometacarpal area or dorsum of hand; without free graft. ( **I** ) Arthroplasty, interposition, intercarpal or carpometacarpal joints. ( **J** ) Repair or advancement, flexor tendon, not in zone 2 primary or secondary; without free graft.

A total of 10 CPT codes accounting for 27,986 hand surgical procedures were reviewed. Corresponding demographic data are shown in Supplemental Table S4 . Approximately 4.8% ( *n* = 1,315) of these were performed by plastic surgeons and 94.4% ( *n* = 25,900) by orthopedic surgeons. Plastic surgical involvement comprised 32.4% of soft tissue procedures (suture of digital nerve, tendon repairs or transfers) and 3.6% of bony procedures (fractures, intercarpal or carpometacarpal procedures). Case volumes over the years are shown in Fig. 2 . In the years 2010 and 2020, plastic surgical involvement did not differ significantly, with 5.4% versus 4.3% of hand procedures being performed by plastic surgeons, respectively ( *p* = 0.23). Yearly procedure volume increased for all bony procedures over the study period. This trend was similar for soft tissue procedures until 2018.

Table 3 presents median operative times for each procedure. Comparing plastic surgeons with orthopedic surgeons, the median operative time in minutes were 68 versus 64 for intercarpal or carpometacarpal arthroplasty procedures ( *p* < 0.01), 92 versus 62 and 74 for general surgery for distal radial fracture fixation ( *p* < 0.01), 89 versus 65 for distal radial fracture fixation, two fragments ( *p* < 0.01), 100 versus 68 for both general and orthopedic surgery for distal radial fracture fixation, three or more fragments ( *p* < 0.01), 59 versus 81 for flexor tendon repair ( *p* < 0.01), and 45 versus 55 for suture of digital nerve ( *p* < 0.01).

**Table 3:** Median operative times for selected hand procedures compared across surgical specialties

Procedure	Specialty	Median time (minutes)	Interquartile range	*P* -value ^a^
**Repair, tendon or muscle, flexor, forearm and/or wrist; primary, single, each tendon or muscle**	Plastic surgery	60	48–89	NS
	Orthopedic surgery	62	41–90	
**Tendon transplantation or transfer, flexor or extensor, forearm and/or wrist, single; each tendon**	Plastic surgery	84	45–126	NS
	Orthopedic surgery	70	48–105	
**Arthroplasty, interposition, intercarpal or carpometacarpal joints**	Plastic surgery	68	53–89	<0.01
	Orthopedic surgery	64	46–84	
**Open treatment of distal radial extra-articular fracture or epiphyseal separation, with internal fixation**	Plastic surgery	92	66–109	<0.01 for all comparisons
	General surgery	74	51–95	
	Orthopedic surgery	62	47–82	
**Open treatment of distal radial intra-articular fracture or epiphyseal separation; with internal fixation of two fragments**	Plastic surgery	89	69–116	<0.01
	Orthopedic surgery	65	48–87	
**Open treatment of distal radial intra-articular fracture or epiphyseal separation; with internal fixation of three or more fragments**	Plastic surgery	100	79–128	<0.01 for all comparisons
	General surgery	68	54–98	
	Orthopedic surgery	68	50–91	
**Repair or advancement, flexor tendon, not in zone 2 digital flexor tendon sheath; primary or secondary without free graft, each tendon**	Plastic surgery	59	40–82	<0.01
	Orthopedic surgery	81	57–107	
**Transfer or transplant of tendon, carpometacarpal area or dorsum of hand; without free graft, each tendon**	Plastic surgery	56	40–81	NS
	Orthopedic surgery	55	40–76	
**Transfer or transplant of tendon, palmar; without free tendon graft, each tendon**	Plastic surgery	67	48–103	NS
	Orthopedic surgery	63	48–82	
**Suture of digital nerve, hand or foot; one nerve**	Plastic surgery	45	30–59	<0.01
	Orthopedic surgery	55	37–78	

Notes: ^a^ Mann-Whitney U tests were used for the comparison of two specialties.

Kruskal-Wallis tests were used for the comparison of more than two specialties. In this case, pairwise comparison between plastic surgery and other specialties were performed by Wilcoxon rank-sum tests with Bonferroni-Holm method to adjust *p* -values; *p* -values for the pairwise comparisons are reported in cases where significance tests for comparisons between plastic surgery and different specialties are contrasting.

Differences in complication rates between specialties are presented in Table 4 and Supplemental Table S5 . Complication rates for distal radial fracture fixation, three or more fragments as well as intercarpal or carpometacarpal procedures were grossly inversely proportional to the case volumes performed by the specialties that were compared.

**Table 4:** Hand procedures for which there was a significant difference in complication rates between specialties

Procedure	Type of complication	Specialty	*N*	Complication *N* (%)	*P* -value
**Open treatment of distal radial intra-articular fracture or epiphyseal separation; with internal fixation of three or more fragments**	Readmission	Plastic surgery	234	8 (3.4%)	0.013
		Orthopedic surgery	6,557	82 (1.3%)	
		General surgery	41	0 (0%)	
	Reoperation	Plastic surgery	228	7 (3.1%)	<0.01
		Orthopedic surgery	6,416	61 (1.0%)	
		General surgery	40	0 (0%)	
**Arthroplasty, interposition, intercarpal or carpometacarpal joints**	Superficial infection	Plastic surgery	522	18 (3.4%)	<0.01
		General surgery	42	0 (0%)	
		Orthopedic surgery	2,845	12 (0.4%)	

Note: Procedures for which there was no significant difference are not displayed. *N* for readmission and other complications differs slightly as readmission was not a variable collected in 2010.

### Adult Craniofacial/Facial Reconstruction

**Fig. 3:**
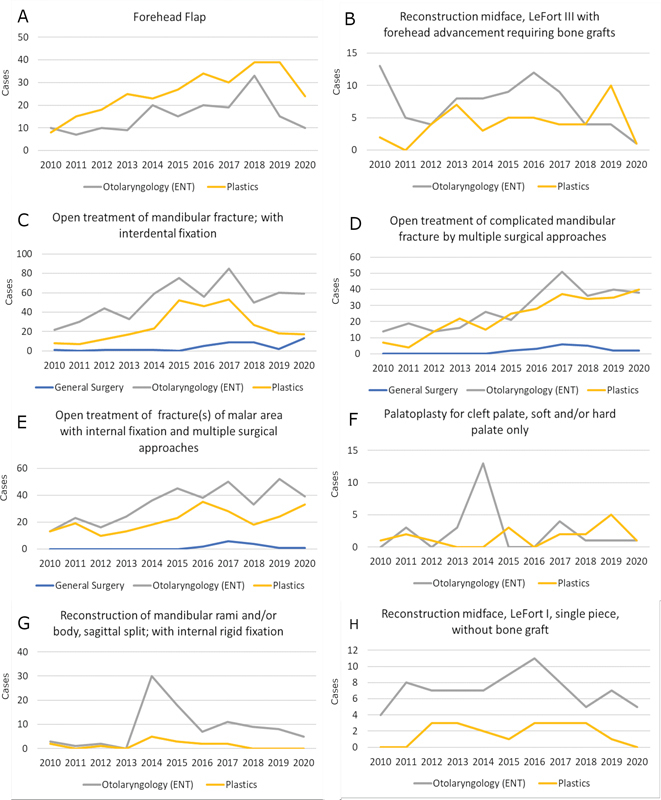
Year on year surgical volumes for selected adult craniofacial/facial reconstructive procedures by operative specialty. The Y axis represents number of cases. ( **A** ) Forehead flap. ( **B** ) Reconstruction of midface, LeFort III with forehead advancement requiring bone grafts. ( **C** ) Open treatment of mandibular fracture, with interdental fixation. ( **D** ) Open treatment of complicated mandibular fracture by multiple surgical approaches. ( **E** ) Open treatment of fracture(s) of malar area with internal fixation and multiple surgical approaches. ( **F** ) Palatoplasty for cleft palate, soft and/or hard palate only. ( **G** ) Reconstruction of mandibular rami and/or body, sagittal split; with internal rigid fixation. ( **H** ) Reconstruction of midface, LeFort I, single piece, without bone fragment.

A total of 13 CPT codes, accounting for 3,026 adult craniofacial and facial reconstructive surgical procedures, were reviewed. Corresponding demographic data and comorbidity analysis are shown in Supplemental Tables S1 and S6 . Five CPT codes were excluded from further analysis due to lack of volume either among plastic surgeons or other specialties. Of the cases reviewed, 89.5% comprised the repair of facial trauma or soft tissue reconstruction ( *n* = 2,707) and 10.5% comprised reconstruction or revision surgery of facial anomalies ( *n* = 319). Plastic surgical involvement constituted 40.7 and 21.6% of facial trauma repairs and reconstruction or revision surgery of facial anomalies, respectively. Case volumes over the years are shown in Fig. 3 . In the years 2010 and 2020, plastic surgeons performed 33.6% versus 39.1% of adult craniofacial procedures ( *p* = 0.28).

Table 5 presents median operative times for each procedure. Comparing plastic surgeons with other specialties, the median operative time in minutes were 93 versus 86 and 149 for otolaryngology and general surgery respectively (NS for otolaryngology; *p* < 0.01 for general surgery) for treatment of complicated zygomatic fractures and 76 versus 94 for otolaryngology ( *p* < 0.01) for forehead flaps. Regarding complications, only wound dehiscence rates in the open treatment of mandible fractures were significantly different with a complication rate of 0.4% for plastic surgeons compared with 3.2% for otolaryngologists ( *p* = 0.04) ( Table 6 and Supplemental Table S7 **)** .

**Table 5:** Median operative times for selected adult craniofacial procedures compared across surgical specialties

Procedure	Specialty	Median time (minutes)	Interquartile range	*P* -value ^a^
**Reconstruction of midface, LeFort I; single piece, segment movement in any direction, without bone graft**	Plastic surgery	126	107–179	NS
	Otolaryngology	143	92–204	
**Reconstruction of mandibular rami and/or body, sagittal split; with internal rigid fixation**	Plastic surgery	135	109–143	NS
	Otolaryngology	114	73–177	
**Open treatment of depressed malar fracture, including zygomatic arch and malar tripod**	Plastic surgery	44	19–92	NS
	Otolaryngology	63	36–100	
**Open treatment of complicated fracture(s) of malar area, including zygomatic arch and malar tripod; with internal fixation and multiple surgical approaches**	Plastic surgery	93	57–136	NS for otolaryngology, <0.01 for general surgery
	Otolaryngology	86	55–126	
	General surgery	149	84–165	
**Open treatment of mandibular fracture, with interdental fixation**	Plastic surgery	112	77–151	NS
	Otolaryngology	104	70–148	
	General surgery	126	98–165	
**Open treatment of complicated mandibular fracture by multiple surgical approaches including internal fixation, interdental fixation, and/or wiring of dentures or splints**	Plastic surgery	121	85–185	NS
	Otolaryngology	135	98–197	
	General surgery	117	100–180	
**Palatoplasty for cleft palate, soft and/or hard palate only**	Plastic surgery	65	59–86	NS
	Otolaryngology	71	36–109	
**Forehead flap with preservation of vascular pedicle**	Plastic surgery	76	55–120	0.01
	Otolaryngology	94	63–132	

Notes: ^a^ Mann-Whitney U tests were used for the comparison of two specialties.

Kruskal-Wallis tests were used for the comparison of more than two specialties. In this case, pairwise comparison between plastic surgery and other specialties were performed by Wilcoxon rank-sum tests with Bonferroni-Holm method to adjust *p* -values; *p* -values for the pairwise comparisons are reported in cases where significance tests for comparisons between plastic surgery and different specialties are contrasting.

**Table 6:** Craniofacial/facial reconstruction procedures for which there was a significant difference in complication rates between specialties

Procedure	Type of complication	Specialty	*N*	Complication, *N* (%)	*P* -value
**Open treatment of complicated mandibular fracture by multiple surgical approaches**	Dehiscence	Plastic surgery	261	1 (0.4%)	0.036
		General surgery	20	0 (0%)	
		Otolaryngology	311	10 (3.2%)	

Note: Procedures for which there was no significant difference are not displayed. *N* for readmission and other complications differs slightly as readmission was not a variable collected in 2010.

### Ventral Hernia

**Fig. 4:**
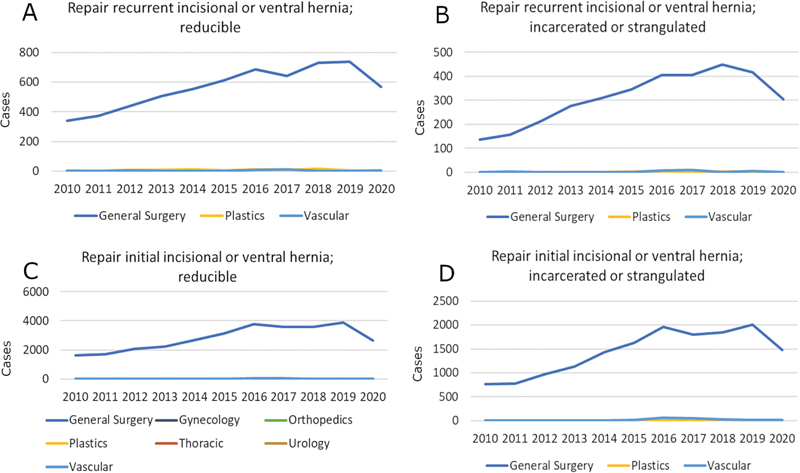
Year on year surgical volumes for selected ventral hernia procedures by operative specialty. The Y axis represents number of cases. ( **A** ) Repair recurrent incisional or ventral hernia; reducible. ( **B** ) Repair recurrent incisional or ventral hernia; incarcerated or strangulated. ( **C** ) Repair initial incisional or ventral hernia; reducible. ( **D** ) Repair initial incisional or ventral hernia; incarcerated or strangulated.

Four CPT codes, accounting for 57,324 open ventral hernia repair procedures, were reviewed. Demographic data for these procedures are shown in Supplemental Table S8 . Over the period studied, general surgery performed 98.2% of the procedures ( *n* = 56,305) with increasing case volumes over the years. By contrast, plastic surgeons performed 0.6% of the cases ( *n* = 326) with steady case volumes over the study period ( Fig. 4 ).

Comparing plastic surgeons with general surgeons, the median operative times in minutes were 138 versus 51 for reducible ventral hernia repair ( *p* < 0.01), 115 versus 49 for incarcerated/strangulated ventral hernia repair ( *p* < 0.01), 132 versus 74 for recurrent ventral hernia repair ( *p* < 0.01), and 184 versus 75 for recurrent and incarcerated/strangulated ventral hernia repair ( *p* < 0.01) ( Table 7 ). Comparisons to other specialties were also significant with results in Table 7 .

**Table 7:** Median operative times for selected ventral hernia procedures compared across surgical specialties

Procedure	Specialty	Median time (minutes)	Interquartile range	*P* -value ^a^
**Repair initial incisional or ventral hernia; reducible**	Plastic surgery	138	95–182	<0.01 for all comparisons
	Urology	72	44–116	
	General surgery	51	31–88	
	Ob/Gyn	63	44–87	
	Vascular surgery	62	39–108	
**Repair initial incisional or ventral hernia; incarcerated or strangulated**	Plastic surgery	115	64–162	<0.01 for all comparisons
	General surgery	49	31–79	
	Ob/Gyn	82	41–152	
	Vascular surgery	54	38–75	
**Repair recurrent incisional or ventral hernia; reducible**	Plastic surgery	132	95–196	<0.01 for all comparisons
	General surgery	74	44–124	
	Vascular surgery	78	56–123	
**Repair recurrent incisional or ventral hernia; incarcerated or strangulated**	Plastic surgery	184	147–246	<0.01 for all comparisons
	General surgery	75	45–122	
	Vascular surgery	68	52–77	

Notes: ^a^ Mann-Whitney U tests were used for the comparison of two specialties.

Kruskal-Wallis tests were used for the comparison of more than two specialties. In this case, pairwise comparison between plastic surgery and other specialties were performed by Wilcoxon rank-sum tests with Bonferroni-Holm method to adjust *p* -values; *p* -values for the pairwise comparisons are reported in cases where significance tests for comparisons between plastic surgery and different specialties are contrasting.

In general, complication rates for each included specialty tended to be inversely proportional to case volumes; these are shown in Table 8 and Supplemental Table S9 .

**Table 8:** Ventral hernia procedures for which there was a significant difference in complication rates between specialties

Procedure	Type of complication	Specialty	*N*	Complication, *N* (%)	*P* -value
**Repair initial incisional or ventral hernia; reducible**	Readmission	Plastic surgery	204	13 (6.4%)	<0.01
		General surgery	29,273	1157 (4.0%)	
		Ob/Gyn	63	9 (14%)	
		Orthopedic surgery	13	1 (8%)	
		Thoracic	40	1 (2.5%)	
		Urology	39	4 (10.2%)	
		Vascular	189	7 (3.7%)	
	DVT	Plastic surgery	210	3 (1.4%)	0.026
		General surgery	30,868	59 (0.2%)	
		Ob/Gyn	64	0 (0%)	
		Orthopedic surgery	13	0 (0%)	
		Thoracic	41	0 (0%)	
		Urology	40	0 (0%)	
		Vascular	194	0 (0%)	
**Repair recurrent incisional or ventral hernia; reducible**	DVT	Plastic surgery	84	2 (2.4%)	<0.01
		General surgery	6179	16 (0.3%)	
		Vascular	42	0 (0%)	
**Repair recurrent incisional or ventral hernia; incarcerated or strangulated**	Pulmonary embolism	Plastic surgery	16	1 (5.9%)	0.027
		General surgery	3,415	22 (0.6%)	
		Vascular	26	0 (0%)	

Note: Procedures for which there was no significant difference are not displayed. *N* for readmission and other complications differs slightly as readmission was not a variable collected in 2010.

### Peripheral Nerve

**Fig. 5:**
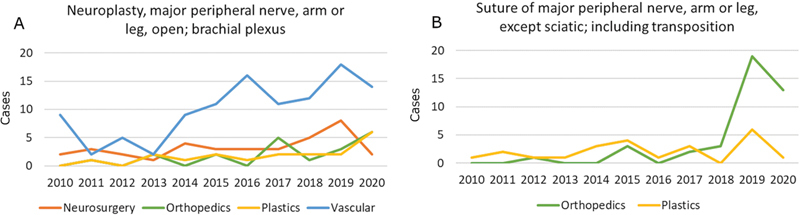
Year on year surgical volumes for selected peripheral nerve procedures by operative specialty. The Y axis represents number of cases. ( **A** ) Neuroplasty, major peripheral nerve, arm or leg, open; brachial plexus. ( **B** ) Suture of major peripheral nerve, arm or leg, except sciatic, including transposition.

Five CPT codes, accounting for 317 peripheral nerve procedures, were reviewed. Corresponding demographic data are shown in Supplemental Table S10 . Three procedures were excluded from further analysis due to less than 10 cases performed by one of the specialties that were compared. Approximately 21.1% of all reviewed procedures were performed by plastic surgeons ( *n* = 67). Moreover, 57.1 and 18.8% of brachial plexus neuroplasty procedures were performed by vascular surgeons and neurosurgeons, respectively **(** Fig. 5 ).

There were no significant differences between plastic surgery and other specialties in either operative time or complications ( Table 9 and Supplemental Table S11 **)** .

**Table 9:** Median operative times for selected peripheral nerve procedures compared across surgical specialties

Procedure	Specialty	Median time (minutes)	Interquartile range	*P* -value ^a^
**Neuroplasty, major peripheral nerve, arm or leg, open; brachial plexus**	Plastic surgery	137	88–266	NS for all comparisons
	Neurosurgery	146	101–220	
	Orthopedics	109	51–151	
	Vascular surgery	147	113–176	
**Suture of major peripheral nerve, arm or leg, except sciatic, including transposition**	Plastic surgery	73	40–128	NS
	Orthopedics	50	41–92	

Notes: ^a^ Mann-Whitney U tests were used for the comparison of two specialties.

Kruskal-Wallis tests were used for the comparison of more than two specialties. In this case, pairwise comparison between plastic surgery and other specialties were performed by Wilcoxon rank-sum tests with Bonferroni-Holm method to adjust *p* -values; *p* -values for the pairwise comparisons are reported in cases where significance tests for comparisons between plastic surgery and different specialties are contrasting.

### Microsurgery

**Fig. 6:**
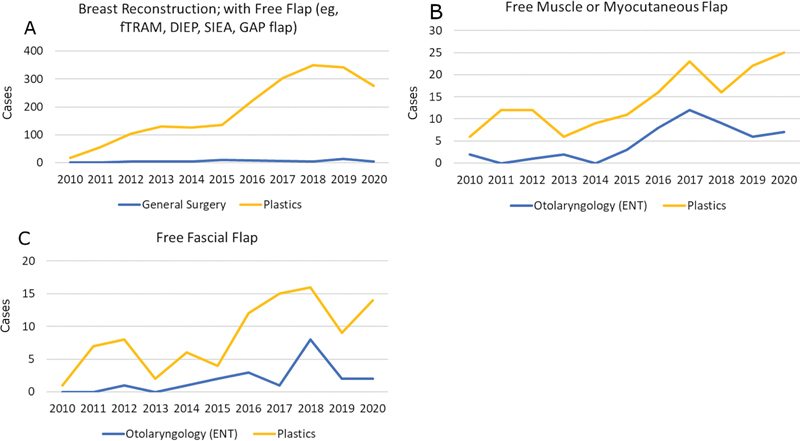
Year on year surgical volumes for selected microsurgical procedures by operative specialty. The Y axis represents number of cases. ( **A** ) Breast reconstruction with free flap (e.g., fTRAM, free transverse rectus abdominus myocutaneous flap; DIEP, deep inferior epigastric perforator flap; SIEA, superficial inferior epigastric artery flap; GAP, gluteal artery perforator flap). ( **B** ) Free muscle or myocutaneous flap. ( **C** ) Free fascial flap.

Three CPT codes accounting for 2,469 microsurgical procedures were reviewed. Corresponding demographic data and comorbidity analysis are shown in Supplemental Tables S1 and S12 . Plastic surgeons performed 97.0% free tissue transfers for breast reconstruction ( *n* = 2062) as compared with 2.9% that were performed by general surgeons ( *n* = 61). Approximately 73.3% of non-breast free tissue transfers were performed by plastic surgeons ( *n* = 252) as compared with 20.3% that were performed by otolaryngologists ( *n* = 70) ( Fig. 6 ). In the years 2010 and 2020, plastic surgeons performed 89.3% versus 94.6% of selected microsurgical procedures ( *p* = 0.25).

Comparing plastic surgeons with otolaryngologists, the median operative time in minutes were 422 versus 487 ( *p* = 0.03) for free muscle or myocutaneous flaps and 329 versus 448 ( *p* = 0.04) for free fascial flaps ( Table 10 ). Regarding complications, the only significant difference was bleeding requiring transfusion following breast free tissue transfers, with complication rates of 15 and 6.6% for general surgeons and plastic surgeons, respectively ( *p* = 0.01) ( Table 11 and Supplemental Table S13 **)** .

**Table 10:** Median operative times for selected microsurgical procedures compared across surgical specialties

Procedure	Specialty	Median time (minutes)	Interquartile range	*P* -value ^a^
**Breast reconstruction with free flap**	Plastic surgery	446	347–567	NS
	General surgery	489	397–588	
**Free muscle or myocutaneous flap with microvascular anastomosis**	Plastic surgery	422	316–531	0.03
	Otolaryngology	487	382–594	
**Free fascial flap with microvascular anastomosis**	Plastic surgery	329	243–476	0.04
	Otolaryngology	448	416–192	

Notes: ^a^ Mann-Whitney U tests were used for the comparison of two specialties.

Kruskal-Wallis tests were used for the comparison of more than two specialties. In this case, pairwise comparison between plastic surgery and other specialties were performed by Wilcoxon rank-sum tests with Bonferroni-Holm method to adjust *p* -values; *p* -values for the pairwise comparisons are reported in cases where significance tests for comparisons between plastic surgery and different specialties are contrasting.

**Table 11:** Microsurgical procedures for which there was a significant difference in complication rates between specialties

Procedure	Type of complication	Specialty	*N*	Complication, *N* (%)	*P* -value
**Breast reconstruction with free flap (e.g., fTRAM, DIEP, SIEA, GAP flap)**	Bleed requiring transfusion	Plastic surgery	2,058	135 (6.6%)	0.01
		General surgery	60	9 (15%)	

Note: Procedures for which there was no significant difference are not displayed. *N* for readmission and other complications differs slightly as readmission was not a variable collected in 2010.

## Discussion

This study serves as a review of case volumes, operative times, and complication rates of select procedures that are performed by both plastic surgeons and surgeons of other specialties over a recent 10-year span in the ACS-NSQIP. We have examined whether plastic surgeons continue to perform common reconstructive breast, hand, adult craniofacial, ventral hernia, peripheral nerve, and microsurgery procedures in meaningful numbers and whether patient outcomes are associated with case volumes. A condensed summary of the results can be found in Table 12 . An analysis of rates of smoking, diabetes, and average BMI was performed with significant differences between specialties only found for three CPT codes ( Supplemental Table S1 ).

**Table 12:** A condensed summary of results

Area of practice	General trends	Operative time findings	Complication findings
Breast reconstruction	• Overall increase in volumes with plastic surgeons performing majority of cases	• Plastic surgeons had shorter operative times with the exception of gynecomastia excision	• Overall lower complication rates for plastic surgeons
Hand surgery	• Overall increase in case volumes • Orthopedic surgeons perform a majority of cases • Plastic surgeons perform a larger proportion of soft tissue cases than bony cases	• Plastic surgeons had longer operative times with the exception of soft tissue cases	• Overall higher complication rates for plastic surgeons
Adult craniofacial surgery	• Plastic surgeons had an overall increase in the proportion of cases performed from 2010 to 2020	• Largely no significant differences for operative times with the exception of two cases where plastic surgeons had shorter times	• Largely no significant differences with the exception of one case where plastic surgeons had lower complication rates
Ventral hernia repair	• Plastic surgeons perform a very small proportion of ventral hernia repairs compared to general surgeons	• Plastic surgeons have consistently longer operative times compared to general surgeons	• Plastic surgeons have consistently higher complication rates compared to general surgeons
Peripheral nerve	• Plastic surgeons performed approximately one-fifth of peripheral nerve cases	• No significant differences in operative times	• No significant differences in complication rates
Microsurgery	• Plastic surgeons consistently perform the highest volume of microsurgical cases among CPT codes analyzed	• Plastic surgeons had lower operative times for cases analyzed	• Complication rates only differed for one complication in one set of cases in which plastic surgeons had lower bleed rates than general surgeons

Our findings demonstrate that plastic surgeons continue to perform an overwhelming majority of reconstructive breast and microsurgery procedures. Although other areas of interest were more commonly performed by other surgical specialties, the proportion of cases performed by plastic surgeons did not significantly decrease over the study period for any of these. Moreover, as a general rule, complication rates and operative times varied between specialties and were inversely proportional to case volumes. Although operative times longer than a certain cutoff value have previously been associated with postoperative complications in specific clinical scenarios, 11 12 our findings are not sufficient to show such associations.

Reconstructive breast procedures, a traditional area of focus for plastic surgeons, have remained so both in terms of volume and performance metrics. All selected breast procedures were mainly performed by plastic surgeons with lower operative times and complication rates except gynecomastia excision, which was mainly performed by general surgeons. As mentioned above, case volume discrepancies might explain the differences in lower operative times and complication rates.

Modern hand surgery has deep multidisciplinary roots in the plastic and orthopedic surgical communities. In contemporary hand practice, there are greater numbers of orthopedic providers as compared with plastic surgery providers. In the first decade of the 21st century there were almost four times greater hand fellows with an orthopedic background as compared with a plastic surgical. 13 Perhaps unsurprisingly, this is reflected in our findings with orthopedic surgeons performing the majority of every reviewed procedure. Interestingly, the difference of involvement by orthopedic versus plastic surgery was smaller for soft tissue procedures than for bony procedures. These soft tissue procedures were the only ones that plastic surgeons performed significantly faster. With regards to complications the differences in complication rates for intercarpal or carpometacarpal procedures as well as distal radial fracture fixation of three or more fragments were inversely proportional to the case volumes.

Facial trauma is an area of shared responsibility between plastic surgeons, otolaryngologists, and oral surgeons, with facial trauma call often shared by these specialties at many institutions. Oral surgery is currently not included as a specialty in ACS-NSQIP; therefore, it was not included in the analyses. Our findings demonstrated similar case volumes for included procedures among otolaryngology and plastic surgery. In general, there were few differences in operative times and complication rates for otolaryngology and plastic surgery with otolaryngologists performing faster zygoma fracture fixations and plastic surgeons having lower operative times for forehead flaps as well as lower rates of wound dehiscence for mandibular fracture repairs.

Although the component separation approach for ventral hernias was developed by the plastic surgeon Oscar M. Ramirez, 5 fewer plastic surgeons enter the specialty via the independent pathway and obtain adequate exposure to intra-abdominal procedures, even for open ventral hernia repair as analyzed here. As such, most of the ventral hernia repairs were found to be performed by general surgeons with significantly shorter operative times and lower complication rates. On the other hand, involvement of plastic surgeons did not significantly differ between the years 2010 and 2020. Although the component separation was an important historical milestone in hernia repair, refined novel techniques such as posterior component separations have been developed by minimally invasive surgeons. Since ACS-NSQIP does not include metrics for case complexity for ventral hernia repairs, no further conclusions could be made.

Relative to other areas of practice, peripheral nerve procedures were fewer in number both in total and among plastic surgeons. Vascular surgeons, who commonly perform first rib resections and scalenectomies, accounted for the highest volume of brachial plexus neuroplasties while nerve repairs were more frequently performed by plastic and orthopedic surgeons. Due to the low case numbers, no conclusions could be drawn regarding operative times or complication rates. Additionally, CPT codes for neuroplasty do not distinguish between the types of procedures typically performed by vascular surgeons as compared with plastic and orthopedic surgeons.

Plastic surgeons continue to contribute to microsurgery, as demonstrated by the increasing microsurgical case volumes, both in general and by plastic surgeons. Plastic surgeons continued to perform the majority of microsurgical breast reconstruction. On the other hand, case volumes of non-breast free tissue transfers performed by otolaryngologists have also increased during the study period. This is likely due to the evolution of the head and neck surgeon into both the extirpative and reconstructive surgeon. 14 15 16 Free flap reconstruction is variable by nature, resulting in a wide range of operative times. Although operative times for both muscular/myocutaneous and fasciocutaneous flaps by plastic surgeons were found to be lower, only limited conclusions can be drawn from this as otolaryngologists do not normally perform reconstruction outside of the head and neck unlike plastic surgeons.

### Limitations

There are multiple limitations of this study. Cases in ACS-NSQIP are meant to be a representative subset of surgeries performed across the United States; this confines the generalizability of our findings to a single healthcare system. The areas of selected procedures are also not comprehensive. This is for multiple reasons; first, the ACS-NSQIP only includes 12 specialties with notable exclusions including dermatology or oral surgery, making comparisons relating to skin cancer surgery or maxillofacial surgery difficult. Relatedly, the NSQIP database does not collect additional information regarding surgeon training including fellowship or cross-specialty training. Second, only adult patients are included; therefore, we cannot make any conclusions regarding pediatric hand or craniofacial procedures. Similarly, socioeconomic data are not included for patients and so it is impossible to account for this. Third, we included CPT codes but not diagnostic codes (e.g., ICD-10 codes); therefore, accurate cross-specialty comparisons for specific diagnoses such as burns could not be made, and this patient group was therefore excluded. Fourth, reviewed procedures were limited to cases with only one CPT code. Complex surgeries involving multiple procedures, such as oncologic resections followed by reconstruction, were excluded to avoid factors that would confound the operative times or complications. Fifth, since plastic surgeons frequently work in conjunction with other services, the numbers included for analysis are naturally lower than the true numbers of procedures. This low number of cases can make cross-specialty comparisons difficult when two specialties are seen to greatly differ in case volume, such as seen between plastic surgery and general surgery for breast reconstruction. Related to this, specialties with under 10 procedures or CPT codes without sufficient total volume were excluded from analysis. This was done to limit the study only to specialties that contribute robustly to practice and procedures that are performed frequently. This has the limitation of excluding rare, but clinically significant, cases. Overall year-by-year volume comparisons were limited to simple trend comparisons due to these considerations and the simplicity of NSQIP data and future studies would benefit from more sophisticated data gathering and analysis. It is also not possible to stratify cases in the ACS-NSQIP database on the basis of complexity. Although the same CPT code is used for all breast free tissue transfers, there is significant variability in case complexity when different free flaps are used, or even on a patient-to-patient basis. Similar examples apply to all the areas examined. Thus, it is difficult to compare the complexity of cases undertaken by one specialty versus another. Furthermore, since it is a pooled dataset, individual characteristics of practitioners or hospitals such as experience level, institutional volumes of surgery, and hospital location are not collected. Additionally, although *p* -value adjustment methods could be beneficial for the comparison of complication rates or frequencies of procedures to reduce the risk of type-I errors, we did not utilize such methods due to the low number of complication events in relevant comparisons, as well as the possible increase in type-II error risk that could be introduced by such methods for the comparisons of procedure frequencies. Finally, since the ACS-NSQIP only includes complications from the first 30 days following surgery, long-term complications and outcomes could not be analyzed.

### Conclusions

Plastic surgeons are trained to operate on various tissues and organs all over the human body. As a result, overlaps with other specialties are inevitable and this invites comparison between specialties regarding case volumes and complications. In those areas where plastic surgeons perform the largest volume of cases, operative times and complication rates were found to be lower compared with other surgical specialties. Those areas in which plastic surgeons have more complications and longer operative times represent opportunities for quality improvement by cross-specialty trainee education. Two easily identifiable areas are hand surgery and ventral hernia surgery, in which expanded trainee rotations would provide more robust education. Additionally, although more detailed analysis is necessary, these data suggest that hospitals should closely examine the relationship between volumes and outcomes across specialties and the relationship of these with referral systems. Plastic surgeons should continue to direct efforts toward ongoing collaboration. Such collaborations have already given rise to multi-disciplinary approaches in many areas including limb salvage, advanced prosthetics, and transgender care. 17 18

## References

[1] McGregor I A Morgan G Axial and random pattern flaps Br J Plast Surg 1973 26 03 202 213 4580012 10.1016/0007-1226(73)90003-9

[2] Taylor G I Daniel R K The free flap: composite tissue transfer by vascular anastomosis Aust N Z J Surg 1973 43 01 1 3 4200573 10.1111/j.1445-2197.1973.tb05659.x

[3] Daniel R K Taylor G I Distant transfer of an island flap by microvascular anastomoses. A clinical technique Plast Reconstr Surg 1973 52 02 111 117 4578998 10.1097/00006534-197308000-00001

[4] Taylor G I Palmer J H The vascular territories (angiosomes) of the body: experimental study and clinical applications Br J Plast Surg 1987 40 02 113 141 3567445 10.1016/0007-1226(87)90185-8

[5] Ramirez O M Ruas E Dellon A L “Components separation” method for closure of abdominal-wall defects: an anatomic and clinical study Plast Reconstr Surg 1990 86 03 519 526 2143588 10.1097/00006534-199009000-00023

[6] Ikuta Y Kubo T Tsuge K Free muscle transplantation by microsurgical technique to treat severe Volkmann's contracture Plast Reconstr Surg 1976 58 04 407 411 959415 10.1097/00006534-197610000-00002

[7] Komatsu S Tamai S Successful replantation of a completely cut-off thumb: case report Plast Reconstr Surg 1968 42 04 374 377

[8] Murray J E Merrill J P Harrison J H Kidney transplantation between seven pairs of identical twins Ann Surg 1958 148 03 343 359 13571912 10.1097/00000658-195809000-00004 PMC1450810

[9] Ono S J The birth of transplantation immunology: the Billingham-Medawar experiments at Birmingham University and University College London. 1951 J Exp Biol 2004 207(Pt 23):4013 4014 15498946 10.1242/jeb.01293

[10] Mackenzie E L Larson J D Poore S O Plastic surgery and specialty creep: an analysis of publication trends Arch Plast Surg 2021 48 06 651 659 34818713 10.5999/aps.2021.00745 PMC8627949

[11] Hardy K L Davis K E Constantine R S The impact of operative time on complications after plastic surgery: a multivariate regression analysis of 1753 cases Aesthet Surg J 2014 34 04 614 622 24696297 10.1177/1090820X14528503

[12] Cheng H Clymer J W Po-Han Chen B Prolonged operative duration is associated with complications: a systematic review and meta-analysis J Surg Res 2018 229 134 144 29936980 10.1016/j.jss.2018.03.022

[13] Higgins J P The diminishing presence of plastic surgeons in hand surgery: a critical analysis Plast Reconstr Surg 2010 125 01 248 260 20048616 10.1097/PRS.0b013e3181c496a2c

[14] Bur A M Villwock M R Nallani R National database research in head and neck reconstructive surgery: a call for increased transparency and reproducibility Otolaryngol Head Neck Surg 2021 164 02 315 321 32633679 10.1177/0194599820938044

[15] Zhang J X Wan M Ding Y Do microsurgical outcomes differ based on which specialty does the operation? A NSQIP analysis Plast Reconstr Surg Glob Open 2020 8 04 e2769 32440436 10.1097/GOX.0000000000002769 PMC7209891

[16] Lee M Haller H S Gosain A K Evolution of practice patterns in plastic surgery using Current Procedural Terminology mapping: a 9-year analysis of cases submitted by primary and recertification candidates to the American Board of Plastic Surgery Plast Reconstr Surg 2015 135 03 631e 637e 25719727 10.1097/PRS.0000000000000975

[17] Nigam M Zolper E G Sharif-Askary B Expanding criteria for limb salvage in comorbid patients with nonhealing wounds: the MedStar Georgetown Protocol and lessons learned after 200 lower extremity free flaps Plast Reconstr Surg 2022 150 01 197 209 35583438 10.1097/PRS.0000000000009236

[18] Coleman E Radix A E Bouman W P Standards of Care for the health of transgender and gender diverse people, Version 8 Int J Transgender Health 2022 23 01 S1 S259 10.1080/26895269.2022.2100644 PMC9553112 36238954

