# Supplementary figures and images for: Coexisting in a Crowded Field: A 10-year Comparison of Procedural Volumes of Plastic Surgeons and Other Surgical Specialties in the United States
Source: Arch Plast Surg. 2026 Jan 30;53(1):102–16. doi: 10.1055/a-2731-4559 (PMC12858312; doi:10.1055/a-2731-4559)

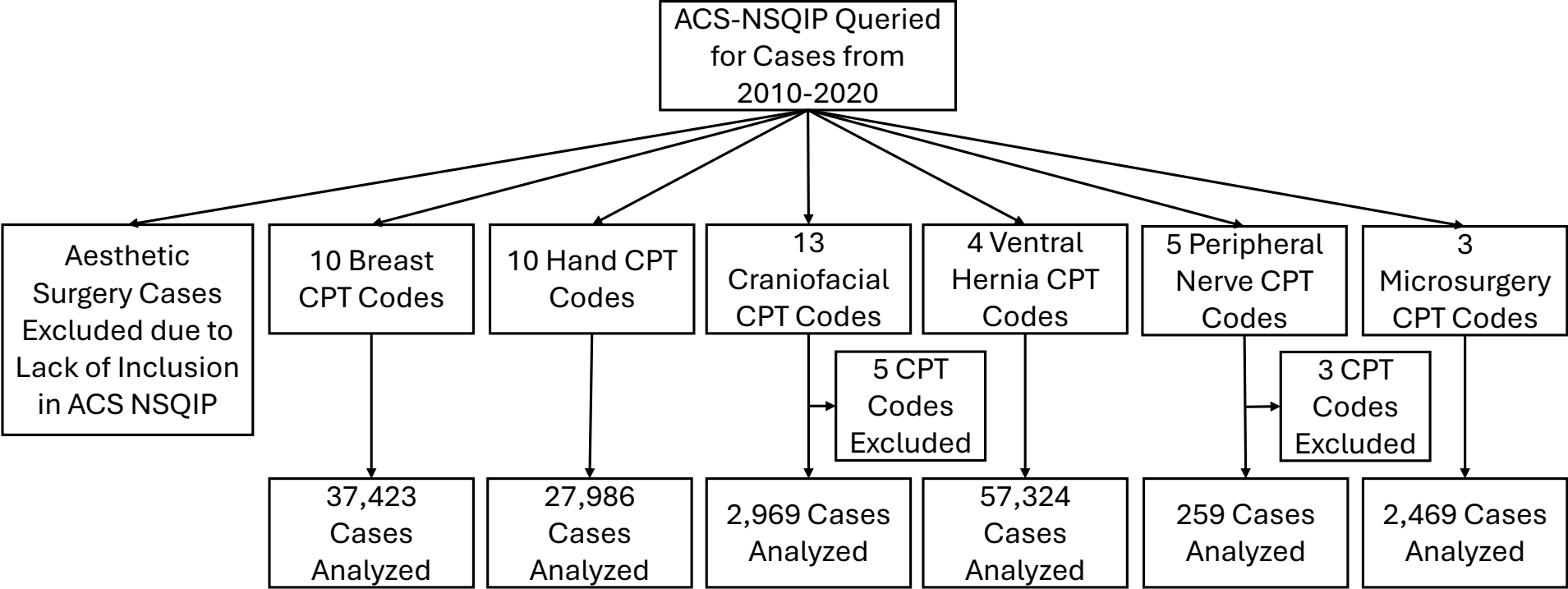

Supplement: Supplementary file 1 — Supplementary Material [file 10-1055-a-2731-4559-s24sep0147oa-1.pdf]
