# Supplementary material for: Coexisting in a Crowded Field: A 10-year Comparison of Procedural Volumes of Plastic Surgeons and Other Surgical Specialties in the United States
Source: Arch Plast Surg. 2026 Jan 30;53(1):102–16. doi: 10.1055/a-2731-4559 (PMC12858312; doi:10.1055/a-2731-4559)
Supplement: Supplementary file 2 — Supplementary Material [file 10-1055-a-2731-4559-s24sep0147oa-2.pdf]

	Mastectomy for Gynecomastia			Free Muscle or Myocutaneous Flap with Microvascular Anastomosis		
	General Surgery (N=2453)	Plastics (N=708)	p value	Otolaryngology (ENT) (N=50)	Plastics (N=158)	p value
**BMI**			0.007			0.014
**Median (Q1, Q3)**	27.122 (24.933, 29.834)	27.612 (24.952, 30.792)		24.201 (19.762, 29.035)	26.579 (22.373, 32.911)	

	Mastectomy for Gynecomastia			Reconstruction of mandibular rami and/or body, sagittal split; with internal rigid fixation		
**Smoking Status**	General Surgery	Plastic Surgery	P-Value	ENT	Plastic Surgery	P-Value
**No**	2088	631	p<0.01	88	8	p<0.01
**Yes**	364	76		6	7	

Supplemental Table 1: Comorbidity analysis. CPT codes for which there was a statistically significant difference between smoking status, diabetes, or median BMI among surgical specialties are shown. Chi-Square test performed for smoking and diabetes status and Kruskal-Wallis for median BMI.

Procedure	CPT Code	N	Mean Age	Sex			Diabetes		Smoking Status	
				Female	Male	Other or No Response	Yes	No	Yes	No
Mastectomy for gynecomastia	19300	3170	34	79	3091	0	97	3073	441	2729
Mastopexy	19316	1228	49	1209	19	0	74	1154	78	1150
Breast reduction	19318	18599	42	18336	257	6	921	17678	1553	17046
Breast augmentation with implant	19325	4196	36	4091	101	4	58	4138	545	3651
Insertion of breast implant on same day of mastectomy	19340	1263	51	1256	7	0	60	1203	100	1163
Insertion or replacement of breast implant on separate day from mastectomy	19342	3009	52	3001	8	0	202	2807	279	2730
Nipple/areola reconstruction	19350	1020	51	1009	10	1	72	948	65	955
Tissue expander placement in breast reconstruction,	19357	3331	51	3320	10	1	247	3084	371	2960
Revision of peri-implant capsule, breast, including capsulotomy, capsulorrhaphy, and/or partial capsulectomy	19370	357	51	355	2	0	22	335	43	314
Peri-implant capsulectomy, breast, complete, including removal of all intracapsular contents	19371	1250	56	1245	5	0	105	1145	134	1116

Supplemental Table 2: Demographic and clinical data of patients for selected breast CPT codes

	Mastectomy for gynecomastia					Mastopexy					Breast reduction				
	General Surgery		Plastics			General Surgery		Plastics			General Surgery		Plastics		
	N	%	N	%	P-Value	N	%	N	%	P-Value	N	%	N	%	P-Value
**Superficial Infection**	36	1.5	6	0.8	0.205	0	0	14	1.2	0.462	20	2.6	430	2.4	0.813
**Wound Dehiscence**	7	0.3	1	0.1	0.502	0	0	5	0.4	0.661	9	1.2	128	0.7	0.674
**Pneumonia**	0	0	0	0	NV	0	0	0	0	NV	0	0	11	0.1	0.971
**Pulmonary Embolism**	0	0	1	0.1	0.63	0	0	1	0.1	0.845	0	0	13	0.1	0.961
**Cardiac Arrest**	1	0	0	0	0.591	0	0	0	0	NV	0	0	2	0	0.999
**Blood Transfusion**	1	0	0	0	0.591	0	0	1	0.1	0.845	0	0	40	0.2	0.753
**DVT**	0	0	1	0.1	0.063	0	0	2	0.2	0.944	0	0	15	0.1	0.999
**Reoperation**	53	2.2	14	2	0.726	2	4.4	12	1	0.081	13	1.7	313	1.8	0.153
**Readmission**	36	1.5	7	1	0.332	1	2.2	17	1.4	0.529	13	1.7	264	1.5	0.214

	Delayed breast implant					Nipple/areola reconstruction					Tissue expander placement				
	General Surgery		Plastics			General Surgery		Plastics			General Surgery		Plastics		
	N	%	N	%	P-Value	N	%	N	%	P-Value	N	%	N	%	P-Value
**Superficial Infection**	0	0	24	0.8	0.434	0	0	4	4	0.708	2	0.7	51	1.7	0.181
**Wound Dehiscence**	0	0	12	0.4	0.581	0	0	2	0.2	0.791	3	1	18	0.6	0.394
**Pneumonia**	0	0	0	0	NV	0	0	0	0	NV	0	0	2	0.1	0.657
**Pulmonary Embolism**	0	0	3	0.1	0.783	0	0	1	0.1	0.852	0	0	3	0.1	0.586
**Cardiac Arrest**	0	0	1	0	0.874	0	0	0	0	NV	0	0	0	0	NV
**Blood Transfusion**	1	1.4	0	0	<0.01	0	0	0	0	NV	1	0.3	5	0.2	0.51

**DVT**	1	1.4	2	0	<0.01	0	0	0	0	NV	0	0	7	0.2	0.673
**Reoperation**	4	5.4	60	2	0.17	0	0	7	0.7	0.791	13	4.4	149	4.9	0.775
**Readmission**	4	5.4	59	2	0.044	0	0	4	0.4	0.797	10	3.4	142	4.7	0.224

	**Breast augmentation with implant**					**Immediate breast implant**					**Revision of peri-implant capsule**				
	General Surgery		Plastics			General Surgery		Plastics			General Surgery		Plastics		
	N	%	N	%	P-Value	N	%	N	%	P-Value	N	%	N	%	P-Value
**Superficial Infection**	0	0	16	0.4	0.634	3	2.2	8	0.7	0.074	1	7.1	2	0.6	<0.01
**Wound Dehiscence**	0	0	4	0.1	0.812	3	2.2	11	1	0.191	0	0	0	0	NV
**Pneumonia**	0	0	0	0	NV	0	0	0	0	NV	0	0	1	0.3	0.839
**Pulmonary Embolism**	0	0	2	0	0.867	0	0	0	0	NV	0	0	1	0.3	0.839
**Cardiac Arrest**	0	0	0	0	NV	0	0	0	0	NV	0	0	1	0.3	0.839
**Blood Transfusion**	0	0	2	0	0.867	1	0.7	0	0	<0.01	0	0	1	0.3	0.839
**DVT**	0	0	2	0	0.867	0	0	0	0	NV	0	0	1	0.3	0.839
**Reoperation**	0	0	49	1.2	0.487	8	6	30	2.7	0.192	0	0	4	1.2	0.81
**Readmission**	2	3.4	24	0.6	0.204	5	3.7	25	2.2	0.281	0	0	3	0.9	0.792

	**Peri-implant capsulectomy**				
	General Surgery		Plastics		
	N	%	N	%	P-Value
**Superficial Infection**	3	3.3	12	1	0.056

**Wound Dehiscence**	2	2.2	5	0.4	0.029
**Pneumonia**	0	0	3	0.3	0.628
**Pulmonary Embolism**	0	0	0	0	NV
**Cardiac Arrest**	0	0	0	0	NV
**Blood Transfusion**	1	1.1	4	0.3	0.271
**DVT**	0	0	1	0.1	0.78
**Reoperation**	1	1.1	23	2	0.324
**Readmission**	1	1.1	26	2.3	0.772

Supplemental Table 3: Numbers and rates of complications in breast procedures that were included in the study between specialties. The numbers for readmission and other complications differ slightly as readmission was not a variable collected in 2010.

Procedure	CPT Code	N	Mean Age	Sex			Diabetes		Smoking Status	
				Female	Male	Other or No Response	Yes	No	Yes	No
Repair, tendon or muscle, flexor, forearm and/or wrist; primary, single, each tendon or muscle	25260	175	37	60	115	0	7	168	69	106
Tendon transplantation or transfer, flexor or extensor, forearm and/or wrist, single; each tendon	25310	295	54	150	145	0	21	274	53	242
Arthroplasty, interposition, intercarpal or carpometacarpal joints	25447	3419	62	2519	900	0	418	3001	442	2977
Open treatment of distal radial extra-articular fracture or epiphyseal separation, with internal fixation	25607	8496	57	6529	1965	2	670	7826	1563	6933
Open treatment of distal radial intra-articular fracture or epiphyseal separation; with internal fixation of 2 fragments	25608	7337	55	5197	2140	0	585	6752	1246	6091
Open treatment of distal radial intra-articular fracture or epiphyseal separation; with internal fixation of 3 or more fragments	25609	6841	57	4973	1866	2	641	6200	1277	5564
Repair or advancement, flexor tendon, not in zone 2 digital flexor tendon sheath (eg, no man's land); primary or secondary without free graft, each tendon	26350	545	40	159	386	0	24	521	143	402
Transfer or transplant of tendon, carpometacarpal area or dorsum of hand; without free graft, each tendon	26480	293	59	179	114	0	29	264	56	237

Transfer or transplant of tendon, palmar; without free tendon graft, each tendon	26485	43	54	21	22	0	3	40	6	37
Suture of digital nerve, hand or foot; 1 nerve	64831	542	40	242	300	0	27	515	120	422

Supplemental Table 4: Demographic and clinical data of patients for selected hand CPT codes.

	Flexor tendon repair wrist/forearm					Forearm/wrist flexor tendon transplantation or transfer					Arthroplasty, intercarpal or carpometacarpal joints				
	Orthopedics		Plastics			Orthopedics		Plastics			Orthopedics		Plastics		
	N	%	N	%	P-Value	N	%	N	%	P-Value	N	%	N	%	P-Value
**Superficial Infection**	2	1.8	0	0	0.31	0	0	0	0	NV	12	0.4	18	3.4	<0.01
**Wound Dehiscence**	0	0	0	0	NV	0	0	0	0	NV	3	0.1	0	0	0.743
**Pneumonia**	0	0	0	0	NV	0	0	0	0	NV	0	0	0	0	NV
**Pulmonary Embolism**	0	0	0	0	NV	0	0	0	0	NV	1	0	0	0	0.906
**Cardiac Arrest**	0	0	0	0	NV	0	0	0	0	NV	0	0	0	0	NV
**Blood Transfusion**	0	0	0	0	NV	0	0	0	0	NV	0	0	0	0	NV
**DVT**	0	0	0	0	NV	0	0	0	0	NV	0	0	0	0	NV
**Reoperation**	0	0	0	0	NV	1	0.4	1	1.9	0.336	9	0	1	0.2	0.937
**Readmission**	0	0	0	0	NV	1	0.4	1	1.9	0.338	16	0.6	4	0.1	0.408

	Open treatment of distal radial intra-articular fracture 3 or more fragments					Flexor tendon repair or advancement, not zone 2					Transfer or transplant of tendon, carpometacarpal area or dorsum of hand				
	Orthopedics		Plastics			Orthopedics		Plastics			Orthopedics		Plastics		
	N	%	N	%	P-Value	N	%	N	%	P-Value	N	%	N	%	P-Value
**Superficial Infection**	17	0.3	1	0.4	0.839	0	0	0	0	NV	1	0.5	2	2.5	0.128
**Wound Dehiscence**	4	0.1	0	0	0.919	1	0.3	0	0	0.765	1	0.5	0	0	0.536
**Pneumonia**	8	0.1	0	0	0.845	0	0	0	0	NV	0	0	0	0	NV
**Pulmonary Embolism**	2	0	0	0	0.959	0	0	0	0	NV	0	0	0	0	NV

**Cardiac Arrest Requiring CPR**	0	0	0	0	NV	0	0	0	0	NV	0	0	0	0	NV
**Blood Transfusion**	4	0.1	0	0	0.919	0	0	0	0	NV	0	0	0	0	NV
**DVT**	1	0	0	0	0.979	0	0	0	0	NV	0	0	0	0	NV
**Reoperation**	61	1.0	7	3.1	<0.01	4	1.1	1	0.6	0.944	2	1	1	1.3	0.627
**Readmission**	82	1.3	8	3.4	0.013	0	0	0	0	NV	2	1	2	2.5	0.186

	**Open treatment of distal radial extra-articular fractur**					**Open treatment of distal radial intra-articular fracture, 2 fragments**					**Transfer or transplant of tendon, palmar**				
	Orthopedics		Plastics			Orthopedics		Plastics			Orthopedics		Plastics		
	N	%	N	%	P-Value	N	%	N	%	P-Value	N	%	N	%	P-Value
**Superficial Infection**	19	0.2	0	0	0.845	12	0.2	0	0	0.914	0	0	1	6.7	0.174
**Wound Dehiscence**	4	0	0	0	0.965	1	0	0	0	0.993	0	0	0	0	NV
**Pneumonia**	14	0.1	0	0	0.884	6	0.1	0	0	0.956	0	0	0	0	NV
**Pulmonary Embolism**	4	0	0	0	0.965	2	0	0	0	0.985	0	0	0	0	NV
**Cardiac Arrest Requiring CPR**	4	0	0	0	0.965	1	0	0	0	0.993	0	0	0	0	NV
**Blood Transfusion**	10	0.1	0	0	0.915	7	0.1	0	0	0.949	0	0	0	0	NV
**DVT**	7	0	0	0	0.94	5	0.1	0	0	0.963	0	0	0	0	NV
**Reoperation**	64	0.8	0	0	0.076	59	0.8	1	1.4	0.01	1	3.7	0	0	0.191
**Readmission**	106	1.3	0	0	0.168	98	1.4	0	0	0.246	0	0	0	0	NV

	Suture of digital nerve				
	Orthopedics		Plastics		
	N	%	N	%	P-Value
**Superficial Infection**	0	0	0	0	NV
**Wound Dehiscence**	0	0	0	0	NV
**Pneumonia**	0	0	0	0	NV
**Pulmonary Embolism**	0	0	0	0	NV
**Cardiac Arrest Requiring CPR**	0	0	0	0	NV
**Blood Transfusion**	0	0	0	0	NV
**DVT**	0	0	0	0	NV
**Reoperation**	0	0	3	1.3	0.59
**Readmission**	0	0	3	1.3	0.509

Supplemental Table 5: Numbers and rates of complications in hand procedures that were included in the study between specialties.

The numbers for readmission and other complications differ slightly as readmission was not a variable collected in 2010.

Procedure	CPT Code	N	Mean Age	Sex			Diabetes		Smoking Status	
				Female	Male	Other or No Response	Yes	No	Yes	No
Reconstruction midface, LeFort I; single piece, segment movement in any direction without bone graft	21141	103	30	41	62	0	4	99	8	95
Reconstruction midface, LeFort I; single piece, segment movement in any direction, requiring bone grafts	21145	21	33	11	10	0	0	21	3	18
Reconstruction of mandibular rami, horizontal, vertical, C, or L osteotomy; without bone graft	21193	10	34	7	3	0	0	10	0	10
Reconstruction of mandibular rami and/or body, sagittal split; with internal rigid fixation	21196	114	32	69	45	0	3	111	14	100
Open treatment of depressed malar fracture	21360	124	42	23	100	1	3	121	38	86
Open treatment of complicated fracture(s) of malar area, including zygomatic arch and malar tripod; with internal fixation and multiple surgical approaches	21365	623	40	134	489	0	26	597	246	377
Open treatment of mandibular fracture; with interdental fixation	21462	905	33	163	742	0	31	874	430	475
Open treatment of complicated mandibular fracture by multiple surgical approaches	21470	597	34	88	509	0	17	580	302	295
Plastic repair of cleft lip/nasal deformity; primary bilateral, 1-stage procedure	40701	3	20	1	2	0	0	3	0	3
Palatoplasty for cleft palate, soft and/or hard palate only	42200	45	36	14	31	0	1	44	3	42

Palatoplasty for cleft palate, with closure of alveolar ridge; soft tissue only	42205	7	52	3	4	0	0	7	2	5
Palatoplasty for cleft palate, with closure of alveolar ridge; with bone graft to alveolar ridge	42210	16	27	7	9	0	1	15	1	15
Forehead flap	15731	458	65	219	239	0	57	401	98	360

Supplemental Table 6: Demographic and clinical data of patients for selected craniofacial/facial reconstruction CPT codes.

	**Reconstruction midface, single piece without bone graft**					**Reconstruction of mandibular rami and/or body**					**Forehead flap**				
	Otolaryngology		Plastics			Otolaryngology		Plastics			Otolaryngology		Plastics		
	N	%	N	%	P-Value	N	%	N	%	P-Value	N	%	N	%	P-Value
**Superficial Infection**	1	1.3	0	0	0.52	6	6.4	0	0	0.314	3	1.8	2	0.7	0.294
**Wound Dehiscence**	0	0	0	0	NV	1	1.1	0	0	0.688	0	0	0	0	NV
**Pneumonia**	0	0	0	0	NV	0	0	0	0	NV	0	0	0	0	NV
**Pulmonary Embolism**	0	0	0	0	NV	0	0	0	0	NV	0	0	0	0	NV
**Cardiac Arrest**	0	0	0	0	NV	0	0	0	0	NV	0	0	0	0	NV
**Blood Transfusion**	0	0	0	0	NV	1	1.1	0	0	0.688	0	0	1	0.4	0.439
**DVT**	1	1.3	0	0	0.246	0	0	0	0	NV	0	0	0	0	NV
**Reoperation**	0	0	0	0	NV	0	0	1	6.7	0.008	3	1.8	6	2.1	0.282
**Readmission**	0	0	0	0	NV	0	0	0	0	NV	10	6	20	7.1	0.886

	**Open treatment of depressed malar fracture**					**Open treatment of complicated mandibular fracture**						
	Otolaryngology		Plastics			General Surgery		Otolaryngology		Plastics		
	N	%	N	%	P-Value	N	%	N	%	N	%	P-Value
**Superficial Infection**	0	0	1	2.2	0.189	0	0	3	1	5	1.9	0.536

**Wound Dehiscence**	0	0	0	0	NV	0	0	10	3.2	1	0.4	0.036
**Pneumonia**	0	0	0	0	NV	0	0	0	0	2	0.8	0.28
**Pulmonary Embolism**	0	0	0	0	NV	0	0	0	0	0	0	NV
**Cardiac Arrest**	0	0	0	0	NV	0	0	0	0	0	0	NV
**Blood Transfusion**	0	0	0	0	NV	0	0	1	0.3	1	0.4	0.958
**DVT**	1	1.3	0	0	0.443	0	0	0	0	0	0	NV
**Reoperation**	2	2.6	1	2.2	0.183	0	0	10	3.2	12	4.6	0.257
**Readmission**	2	2.6	0	0	0.531	1	5	8	2.6	7	2.7	0.759

	**Open treatment of mandibular fracture; with interdental fixation**							**Open treatment of complicated fracture(s) of malar area**						
	General Surgery		Otolaryngology		Plastics			General Surgery		Otolaryngology		Plastics		
	N	%	N	%	N	%	P-Value	N	%	N	%	N	%	P-Value
**Superficial Infection**	2	4.8	8	1.4	3	1.1	0.173	0	0	4	1.1	2	0.9	0.895
**Wound Dehiscence**	0	0	8	1.4	1	0.4	0.287	0	0	1	0.3	0	0	0.714
**Pneumonia**	0	0	1	0.2	0	0	0.754	0	0	1	0.3	0	0	0.714
**Pulmonary Embolism**	0	0	0	0	0	0	NV	0	0	0	0	0	0	NV
**Cardiac Arrest**	0	0	0	0	0	0	NV	0	0	1	0.3	0	0	0.714
**Blood Transfusion**	0	0	1	0.2	0	0	0.754	0	0	0	0	0	0	NV
**DVT**	0	0	0	0	1	0.4	0.334	0	0	1	0.3	0	0	0.714
**Reoperation**	1	2.4	14	2.4	8	2.9	0.871	1	7.1	4	1.1	0	0	0.077
**Readmission**	2	4.8	17	3	8	2.9	0.759	1	7.1	5	1.4	5	2.1	0.0154

	Palatoplasty for cleft palate, soft and/or hard palate only				
	Otolaryngology		Plastics		
	N	%	N	%	P-Value
**Superficial Infection**	0	0	0	0	NV
**Wound Dehiscence**	1	3.8	0	0	0.413
**Pneumonia**	0	0	0	0	NV
**Pulmonary Embolism**	0	0	0	0	NV
**Cardiac Arrest**	0	0	0	0	NV
**Blood Transfusion**	0	0	0	0	NV
**DVT**	0	0	0	0	NV
**Reoperation**	0	0	0	0	0.211
**Readmission**	0	0	1	5.9	0.386

Supplemental Table 7: Numbers and rates of complications in craniofacial procedures that were included in the study between specialties. The numbers for readmission and other complications differ slightly as readmission was not a variable collected in 2010.

Procedure	CPT Code	N	Mean Age	Sex			Diabetes		Smoking Status	
				Female	Male	Other or No Response	Yes	No	Yes	No
Repair initial incisional or ventral hernia; reducible	49560	31495	55	17530	13950	15	4489	27006	5843	25652
Repair initial incisional or ventral hernia; incarcerated or strangulated	49561	16024	56	9722	6290	12	2781	13243	3112	12912
Repair recurrent incisional or ventral hernia; reducible	49565	6338	57	3612	2722	4	1184	5154	1261	5077
Repair recurrent incisional or ventral hernia; incarcerated or strangulated	49566	3467	58	2087	1378	2	776	2691	671	2796

Supplemental Table 8: Demographic and clinical data of patients for selected ventral hernia repair CPT codes.

	Repair initial incisional or ventral hernia; reducible									Repair initial incisional or ventral hernia; incarcerated or strangulated								
	General Surgery		Gynecology		Plastics		Urology			General Surgery		Gynecology		Plastics		Vascular		
	N	%	N	%	N	%	N	%	P-Value	N	%	N	%	N	%	N	%	P-Value
**Superficial Infection**	566	1.8	2	3.1	5	2.4	1	2.5	0.907	378	2.4	0	0	0	0	3	1.5	0.711
**Wound Dehiscence**	122	0.4	1	1.6	2	1	0	0	0.547	71	0.5	0	0	0	0	0	0	0.795
**Pneumonia**	191	0.6	1	1.6	1	0.5	1	2.5	0.723	189	1.2	1	6.7	0	0	0	0	0.094
**Pulmonary Embolism**	47	0.2	0	0	1	0.5	0	0	0.775	47	0.3	0	0	0	0	0	0	0.878
**Cardiac Arrest**	25	0.1	0	0	1	0.5	0	0	0.645	43	0.3	0	0	0	0	0	0	0.892
**Blood Transfusion**	155	0.5	0	0	2	1	0	0	0.853	153	1	0	0	0	0	0	0	0.527
**DVT**	51	0.2	0	0	2	1	0	0	0.026	53	0.3	0	0	0	0	0	0	0.99
**Reoperation**	534	1.7	2	3.1	11	5.2	0	0	0.087	334	2.1	1	6.7	0	0	1	0.5	0.275
**Readmission**	1154	3.7	9	14.1	13	6.2	4	10	<0.01	745	4.7	0	0	0	0	3	1.5	0.209

	Repair recurrent incisional or ventral hernia; reducible							Repair recurrent incisional or ventral hernia; incarcerated or strangulated						
	General Surgery		Plastics		Vascular			General Surgery		Plastics		Vascular		
	N	%	N	%	N	%	P-Value	N	%	N	%	N	%	P-Value
**Superficial Infection**	187	3	2	2.4	1	2.4	0.916	145	4.2	0	0	1	3.8	0.683
**Wound Dehiscence**	52	0.8	0	0	0	0	0.586	29	0.8	1	5.9	0	0	0.074

**Pneumonia**	55	0.9	1	1.2	1	2.4	0.573	53	1.6	0	0	0	0	0.713
**Pulmonary Embolism**	13	0.2	0	0	0	0	0.876	22	0.6	1	5.9	0	0	0.027
**Cardiac Arrest**	9	0.1	0	0	0	0	0.912	10	0.3	0	0	0	0	0.939
**Blood Transfusion**	65	1.1	0	0	1	2.4	0.447	46	1.3	0	0	0	0	0.746
**DVT**	15	0.2	2	2.4	0	0	<0.01	15	0.4	0	0	0	0	0.994
**Reoperation**	176	2.8	0	0	2	4.8	0.455	135	4	2	11.8	1	3.8	0.592
**Readmission**	371	6	2	2.4	0	0	0.238	271	7.9	2	11.8	1	3.8	0.626

Supplemental Table 9: Numbers and rates of complications in ventral hernia procedures that were included in the study between specialties. The numbers for readmission and other complications differ slightly as readmission was not a variable collected in 2010.

Procedure	CPT Code	N	Mean Age	Sex			Diabetes		Smoking Status	
				Female	Male	Other or No Response	Yes	No	Yes	No
Neuroplasty, major peripheral nerve, arm or leg, open; brachial plexus	64713	191	39	110	81	0	13	178	34	157
Suture of major peripheral nerve, arm or leg, except sciatic; including transposition	64856	68	46	18	50	0	10	58	24	44
Suture of major peripheral nerve, arm or leg, except sciatic; without transposition	64857	24	41	7	17	0	3	21	9	15
Suture of; brachial plexus	64861	4	50	2	2	0	1	3	0	4
Nerve pedicle transfer; first stage	64905	30	43	11	19	0	3	27	6	24

Supplemental Table 10: Demographic and clinical data of patients for selected peripheral nerve procedure CPT codes.

	Neuroplasty, major peripheral nerve									Suture of major peripheral nerve				
	Neurosurgery		Orthopedics		Plastics		Vascular			Orthopedics		Plastics		
	N	%	N	%	N	%	N	%	P-Value	N	%	N	%	P-Value
**Superficial Infection**	1	2.8	0	0	0	0	1	0.9	0.702	2	4.9	0	0	0.282
**Wound Dehiscence**	0	0	0	0	0	0	1	0.9	0.875	0	0	0	0	NV
**Pneumonia**	0	0	0	0	0	0	1	0.9	0.875	0	0	0	0	NV
**Pulmonary Embolism**	0	0	0	0	0	0	0	0	NV	0	0	0	0	NV
**Cardiac Arrest**	0	0	0	0	0	0	0	0	NV	0	0	0	0	NV
**Blood Transfusion**	0	0	0	0	0	0	1	0.9	0.875	0	0	0	0	NV
**DVT**	0	0	0	0	0	0	0	0	NV	0	0	0	0	NV
**Reoperation**	1	2.8	0	0	0	0	6	5.5	0.132	0	0	0	0	NV
**Readmission**	0	0	0	0	0	0	6	5.5	0.234	0	0	0	0	NV

Supplemental Table 11: Numbers and rates of complications in nerve procedures that were included in the study between specialties. The numbers for readmission and other complications differ slightly as readmission was not a variable collected in 2010.

Procedure	CPT Code	N	Mean Age	Sex			Diabetes		Smoking Status	
				Female	Male	Other or No Response	Yes	No	Yes	No
Breast reconstruction; with free flap (eg, fTRAM, DIEP, SIEA, GAP flap)	19364	2125	51	2121	4	0	154	1971	146	1979
Free muscle or myocutaneous flap with microvascular anastomosis	15756	222	57	85	137	0	36	186	38	184
Free fascial flap with microvascular anastomosis	15758	122	54	63	59	0	18	104	17	105

Supplemental Table 12: Demographic and clinical data of patients for selected microsurgical procedure CPT codes.

	Free muscle or myocutaneous flap					Free fascial flap					Breast reconstruction with free flap				
	Otolaryngology		Plastics			Otolaryngology		Plastics			General Surgery		Plastics		
	N	%	N	%	P-Value	N	%	N	%	P-Value	N	%	N	%	P-Value
**Superficial Infection**	4	8	11	7	0.805	1	5	4	4.3	0.883	4	6.7	75	3.6	0.223
**Wound Dehiscence**	0	0	3	1.9	0.326	2	10	2	2.1	0.082	2	3.3	45	2.2	0.552
**Pneumonia**	2	4	7	4.4	0.896	0	0	1	1.1	0.643	0	0	11	0.5	0.57
**Pulmonary Embolism**	0	0	1	0.6	0.573	0	0	0	0	NV	0	0	12	0.6	0.553
**Cardiac Arrest**	1	2	0	0	0.075	0	0	0	0	NV	0	0	1	0	0.864
**Blood Transfusion**	12	24	26	16.5	0.229	0	0	6	6.4	0.246	9	15	135	6.6	0.01
**DVT**	2	4	1	0.6	0.082	0	0	0	0	NV	0	0	12	0.6	0.826
**Reoperation**	9	18	29	18.4	0.986	2	10	4	4.3	0.526	8	13.3	217	10.5	0.781
**Readmission**	5	10	11	7	0.381	0	0	6	6.4	0.102	2	3.3	108	5.2	0.551

Supplemental Table 13: Numbers and rates of complications in microsurgery procedures that were included in the study between specialties. The numbers for readmission and other complications differ slightly as readmission was not a variable collected in 2010.
